# The ameliorative effect of bloodletting puncture at hand twelve *Jing*-well points on cerebral edema induced by permanent middle cerebral ischemia via protecting the tight junctions of the blood-brain barrier

**DOI:** 10.1186/s12906-017-1979-6

**Published:** 2017-09-26

**Authors:** Nannan Yu, Zhenguo Wang, Yucen Chen, Juntao Yang, Xuan Lu, Yi Guo, Zelin Chen, Zhifang Xu

**Affiliations:** 1 Acupuncture Research Center, Tianjin University of Traditional Chinese Medicine, Tianjin, 300193 China; 2 Acu-moxibustion and Tuina Department, Tianjin University of Traditional Chinese Medicine, Tianjin, 300193 China; 3 Department of Traditional Chinese Medicine, Xijing Hospital, The Fourth Military Medical University, Xi’an, 710032 People’s Republic of China; 4 Xi’an Encephalopathy Hospital of Traditional Chinese Medicine, Xi’an, 710032 China; 5 Wuhan Hospital of Traditional Chinese Medicine, Wuhan, 430000 China; 6 Institute of Acupuncture and Moxibustion, Tianjin Academy of Traditional Chinese Medicine Affiliated Hospital, Tianjin, 300120 China

**Keywords:** Cerebral edema, blood brain barrier, bloodletting puncture at hand twelve *Jing*-well points, tight junction, permanent middle cerebral artery occlusion

## Abstract

**Background:**

Cerebral edema, erupting simultaneously with severe ischemic stroke, might lead to increased intracranial pressure, cerebral herniation, and ultimately death. Studies conducted previously by our team have demonstrated the fact that bloodletting puncture at hand twelve *Jing*-well points (HTWP) could alleviate cerebral edema, which mainly results from the disruption of blood-brain barrier (BBB). The study, therefore, was first designed to demonstrate whether BBB-protection serves an important role in the edema-relief effect of HTWP bloodletting, based on which to research the molecular mechanism underlying.

**Methods:**

The rats were made into model suffering from permanent middle cerebral artery occlusion (pMCAO) and then bloodletting puncture were treated at HTWP once a day. Wet and dry weight method was adopted to evaluate the degree of brain edema, evans blue extravasation and electron microscopy were used to evaluate the integrity of the BBB, and RT-qPCR was carried out to analyze the expression level of occludin, claudin-5, ICAM-1, and VEGF.

**Results:**

Results revealed that bloodletting puncture treatment could reduce water content of brain and the permeability of BBB caused by ischemic stroke. In bloodletting puncture group, ameliorated tight junctions could be observed under electron microscopy. It was demonstrated in further study that, in bloodletting group, compared with pMCAO one, the expression levels of occludin and claudin-5 were up-regulated, while ICAM-1 and VEGF were down-regulated.

**Conclusions:**

In conclusion, bloodletting puncture at HTWP might play a significant role in protecting the tight junctions of BBB, thus alleviating cerebral edema induced by ischemic stroke. Therefore, the therapy of bloodletting puncture at HTWP may be a promising strategy for acute ischemic stroke in the future.

## Background

Ischemic stroke is a destructive cerebrovascular disease and a leading cause of death and disability worldwide [1]. Until now, tissue plasminogen activator (tPA) is the only effective thrombolytic therapy for ischemic stroke, but its efficacy and safety are limited by its narrow treatment time window and side effects [2, 3]. In light of few effective therapeutic strategies and the devastating impacts and social burden of ischemic stroke, it is extremely urgent to develop effective and safe neuroprotective agents.

Cerebral edema, serving as one of severe complications of ischemic stroke, can exacerbate brain injury and induce clinical deterioration [4]. Brain edema consists of two types: cytotoxic and vasogenic. Among the main causes of vasogenic brain edema, the destroy of blood-brain barrier (BBB) can’t be ignored, in which condition, intravascular proteins and fluid are easy to penetrate into the cerebral parenchymal extracellular space, thus leading to vasogenic cerebral edema and reducing the amount of blood flowing to neurons and, finally, resulting in irreversible apoptosis [5, 6]. It is acknowledged that endothelial layer and the tight junctions are primarily responsible for the regulation of BBB permeability, through which blood-sourced hydrophilic molecules flow into brain parenchyma.

Bloodletting puncture at hand twelve *Jing*-well points (HTWP) has been applied as one of the first aid measures in various types of emergency for more than 3000 years in ancient China. Previous studies have shown bloodletting puncture at HTWP may benefit to the conscious distance in acute stroke patients, the neurologic defects and cerebral edema in rat models suffering from central nervous injuries. Two clinical trials [7, 8] and unpublished data conducted among acute stroke patients, bloodletting puncture at *Jing*-well points was proved safe and beneficial to stroke patients distressed by conscious disturbance. Animal studies showed this therapy could reduce infarct size and prolong the survival time, prevent free radical damage, postpone development of hypoxia and acidosis, and inhibit calcium overload in rat brains following permanent middle cerebral artery occlusion (pMCAO) [9, 10]. It is also showed that the therapy could lessen the degree of cerebral edema in pMCAO rats [9, 10]. Despite differences in primary injury, the BBB disruption and pathogenesis overlap between stroke and traumatic brain injury (TBI) [11]. Previous study demonstrated that bloodletting puncture at HTWP could attenuate brain edema in TBI rats as well [12]. It was still, however, unknown whether bloodletting puncture therapy function to regulate BBB to attenuate cerebral edema induced by ischemic stroke.

Hence, in present study, we explored the effect of bloodletting puncture at HTWP on cerebral edema and the BBB permeability using pMCAO rat model. We also investigated whether bloodletting puncture therapy acted on the BBB integrity and the BBB-associated proteins including occludin, claudin-5, ICAM-1 and VEGF.

## Methods

### Animals and experimental groups

All experimental procedures were carried out in strict accordance with the Guidance Suggestions for the Care and Use of Laboratory Animals, formulated by the Ministry of Science and Technology of China. The protocol was approved by the Animal Care and Use Committee of Tianjin University of Traditional Chinese Medicine (Permit Number: TCM-LAEC2017002) and animal handling procedures were performed according with the Tianjin University of Traditional Chinese Medicine guidelines for the care and use of laboratory animals. All surgeries were performed under chloral hydrate anesthesia, and all efforts were made to minimize animal suffering. Dead rats during operation were excluded from the experiments.

**Fig. 1:**
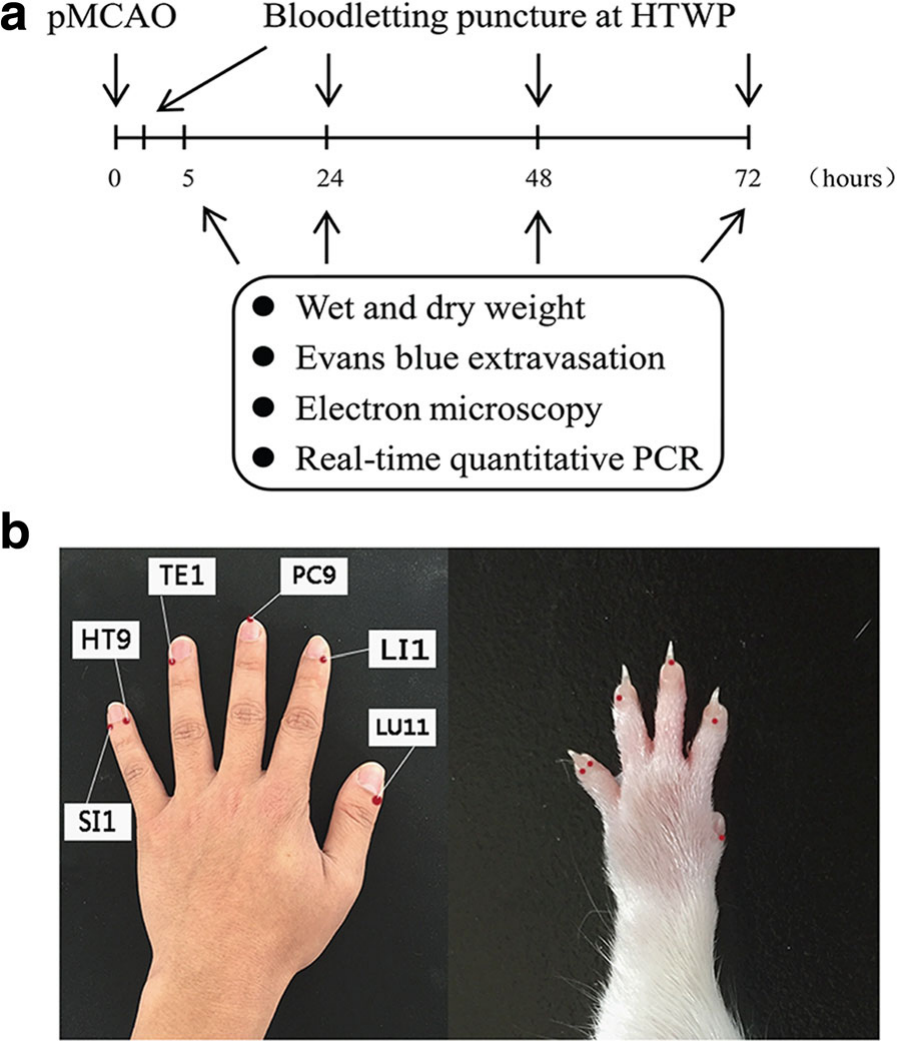
Experimental design. **a** A schematic diagram illustrating the chronological events of experiments. **b** After pMCAO, rats received bloodletting puncture at HTWP once a day. Brain samples were collected at 5 h, 24 h, 48 h, 72 h for wet and dry weight, Evans blue extravasation, electron microscopy and real-time quantitative PCR experiments

Male Wistar rats, weighted 220-250 g, were supplied by the Laboratory Animal Centre of Tianjin University of Traditional Chinese medicine. All rats were allowed to acclimatize for 1 week prior to the experiment, and were housed in a controlled environment (12 h light/dark cycle, 22 ± 2 °C, 55 ± 5% relative humidity) and allowed free access to food and water. All rats were randomly divided into 3groups: sham-operated group, pMCAO group and bloodletting puncture at HTWP + pMCAO group. As shown in Fig. 1a, for the comparative effects of bloodletting on the different durations of ischemia, brain water content was used to assess the brain edema at 24 h, 48 h and 72 h (*n* = 5 per group at each experimental time point). BBB ultrastructure was observed at 48 h (*n* = 3 per group). Evans blue (EB) extravasation were used to assess the degree of BBB integrity at 5 h, 24 h, 72 h after modeling (*n* = 10 per group at each experimental time point). The mRNA expression of claudin-5, occludin, ICAM-1 and VEGF were detected at 5 h, 24 h, 72 h (n = 10 per group at each experimental time point).

### Permanent middle cerebral artery occlusion

A modified Longa method was applied to make permanent middle cerebral artery occlusion (pMCAO) model. Briefly, rats were anesthetized by intraperitoneal injection of 10% chloral hydrate at a dose of 3.5 mL/kg body weight and fixed in a supine position. Then, the right common carotid artery, right external carotid artery, and right internal carotid artery were isolated via a midline incision. Arteries were marked by ligatures on the external carotid artery and the proximal end of the common carotid artery. A silicone-coated nylon monofilament (0.26 mm diameter) was inserted from the distal end of the common carotid artery to the internal carotid artery, about 18.0 ± 0.5 mm in depth to the anterior cerebral artery. Sham-operated control rats underwent the same procedures but without thread insertion. We evaluated the pMCAO rat models successfully established in accordance with a five-point Longa scale [13] after anesthesia recovery. Rats with a score of 1–3 were included and then randomly divided them into model or bloodletting groups. The procedure carried out almost in the third hour.

### Bloodletting puncture at hand twelve *Jing*-well points

At 3 h after pMCAO modeling, a 21 gauge blood lancet (Suzhou Sterilance Medical Equipment Co., Ltd., Suzhou, Jiangsu Province, China) was perpendicularly inserted into skin to a depth of 1 mm in the distal ends of the acupoints bilaterally, with the sequence of LU11 (Shaoshang), LI1 (Shangyang), PC9 (Zhongchong), TE1 (Guanchong), HT9 (Shaochong) and SI1 (Shaoze), to bleed rats in bloodletting group. Comparative anatomy was used for point selection, in reference to human anatomical acupoints [8] shown in Fig. 1b. To bleed 15-20 μl at each acupoint, we squeezed them 3–5 times separately before compressing with cotton balls. And then bloodletting was manipulated at 22 h, 46 h and 70 h. Rats from the control and sham operation groups were grasped with equal strength without bloodletting puncturing at acupoints.

### Measurement of cerebral edema

Cerebral edema was measured by relative water content with wet and dry weight method, which forms as a consequence of BBB breakdown and is calculated as the weight difference between wet and dry samples. Rats (*n* = 5/group) were deeply anesthetized and sacrificed by decapitation at indicated time points (24 h, 48 h, 72 h) after pMCAO. The brains were removed and the ischemic hemisphere was carefully chosen and immediately weighed to obtain the wet weight, dried for 48 h in an oven at 110 °C, and then reweighed to obtain the dry weight. Brain water content was calculated as follows: Brain water content (%) = (wet weight - dry weight) / wet weight × 100%.

### Evaluation of BBB permeability

The BBB permeability was assessed by examining the extent of Evans blue (EB, Sigma, USA) solution leakage from the microvessels in the rat brains following intravenous injection. Briefly, Evans Blue dye (2% in saline, 4 mL/kg) was intravenously administered via tail vein and allowed to circulate for 60 min. To clear the blood and intravascular Evans blue solution that remained in the vascular system, all the rats (*n* = 10 per group at each experimental time point) were deeply anesthetized with 10% chloral hydrate and perfused with heparinized saline through the cardiac ventricle until colorless perfusion fluid was obtained from the atrium at 5 h, 24 h, 72 h after pMCAO. After decapitation, the hemispheres of brain were separated along the interhemispheric plane. For quantitative measurement of EB, the cortex of the ischemic hemispheres were weighed and incubated in formamide (1 ml/100 mg, Amresco) at 54 °C for 24 h and samples were centrifuged at 3000 rpm for 15 min. The supernatant was collected, and the OD at 620 nm was measured using a 722-type spectrophotometer (Shanghai Third Analytical Instrument Factory, Shanghai, China). The amount of Evans Blue in the tissue was quantified using the standard curve. Data was expressed as μg/g of brain tissue.

### Identification of infracted areas after stroke by TTC staining

Rats (*n* = 4–5/group) were deeply anesthetized and sacrificed by decapitation at 48 h after pMCAO, and brains were quickly removed on ice, and placed at −20 °C for 15–20 min. Brains were sectioned coronally into 2 mm slices from an 10-mm-thick brain region which was approximately 2 mm away from the tip of the frontal lobe with a brain matrix (Braintree Scientific, Braintree, MA, USA) on ice. Brain sections were incubated in 2% 2,3,5-triphenyltetrazolium chloride (TTC) (Sigma- Aldrich, Saint Louis, MI, USA) in phosphate buffered saline for 20 min at 37 °C. Finally, the tissues were fixed in 4% paraformaldehyde (in 0.1 M phosphate buffer, pH 7.4). Pictures of the brains sections were documented with a digital camera (Sony, a7r, Japan) and the percentage of cerebral infarct area was calculated with image analysis software (Adobe photoshop CC 2014) according to the formula: [contralateral hemisphere area - (ipsilateral hemisphere area - infarct area)/contralateral hemisphere area] × 100%. Hemispheric swelling was assessed in slices stained by TTC according to the formula: 100% × (ipsilateral volume - contralateral volume)/contralateral volume [14].

### Transmission electron microscopy (TEM)

Transmission electron microscope was performed to study the effects of bloodletting puncture at HTWP on ultrastructural changes of the BBB. At 48 h, 72 h after pMCAO, rats (*n* = 3 per group) were anesthetized with 10% chloral hydrate and perfused with 3% glutaraldehyde and 4% paraformaldehyde in 0.1 M phosphate-buffered saline (PH 7.4). The brains were divided out and the frontal and parietal cortices of ischemic tissues were chosen as samples. The samples were cut into pieces of 1 mm^3^, fixed with 3% glutaraldehyde at 4 °C for 12 h, washed 3 times with PBS buffer. Then the samples were post-fixed in 1% osmium acid for 1 h at 4 °C. After fixation, the samples were dehydrated in grade acetone and embedded in #618 Epon. Ultrathin sections (70 nm) were double stained with uranyl acetate and lead citrate and examined with an TEM (JEM-1011, Japan).

### Real-time quantitative PCR (RT-qPCR)

The cerebral cortex of ischemic penumbra at appointed time were collected and RNA was isolated by using RNAiso Plus (TaKaRa Bio Inc., Shiga, Japan) followed by treatment with DNAs to eliminate contaminating genomic DNA with RNase-free DNase set (TaKaRa). Conventional reverse transcription using 1 μg total RNA with an AffinifyScript QPCR cDNA Synthesis Kit (PrimeScript™ RT reagent Kit with gDNA Eraser, TaKaRa). The cDNA was then used as a template for RT-qPCR, which was performed using the SYBR Premix Ex Taq™ reagent (TaKaRa) and an ABI 7500 system (Applied Biosystems, Foster City, CA, USA) following the manufacturer’s instructions. The gene-specific primer sequences are listed in Table 1. GAPDH was used as internal control for the normalization of gene expression.

**Table 1:** The gene-specific primers used in this study

Gene	Forward primer (5′ → 3′)	Reverse primer (5′ → 3′)	Product size
Occludin	CCGCCAAGGTTCGCTTATCT	TTCAAAAGGCCTCACGGACA	96
Claudin-5	TATGAATCTGTGCTGGCGCT	AGTGCTACCCGTGCCTTAAC	158
ICAM-1	TGTCAGCCACCATGCCTTAG	CAGCTTGCACGACCCTTCTA	132
VEGF-α	ATGCGGATCAAACCTCACCA	AAACCGGGATTTCTTGCGCT	162
GAPDH	AAGAGGGATGCTGCCCTTAC	TACGGCCAAATCCGTTCACA	119

### Statistical analysis

SPSS 16.0 software was used for statistical analysis. All values are expressed as means ± standard deviation (SD). Statistical analyses were performed by using one-way analyses of variance followed by post hoc comparison by using Bonferroni test. A *P* value of less than 0.05 was considered to be statistically significant.

## Results

### Bloodletting puncture at HTWP reduced the brain edema after pMCAO

**Fig. 2:**
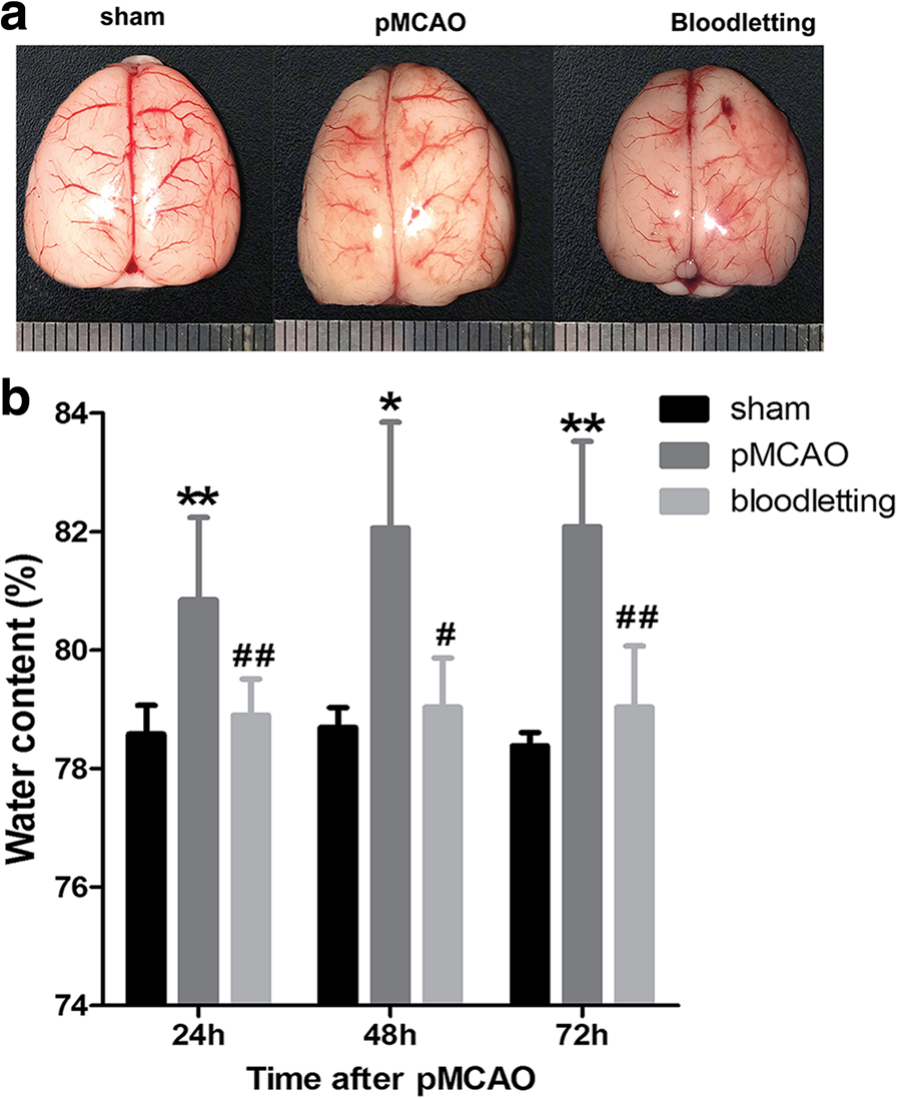
Effect of bloodletting puncture at HTWP on brain edema (*n* = 5 per group). **a** Representative images of brain edema in sham-operated group, pMCAO group, bloodletting group. **b** Quantitative analysis of brain swelling. *P < 0.05, **P < 0.01 vs. sham-operated goup; ^#^ *P* < 0.05, ^##^ *P* < 0.01 vs. pMCAO group at the same time points

Brain water content at 24 h, 48 h and 72 h after ischemia was shown in Fig. 2. The ischemic hemisphere was significantly swelling than that of sham and bloodletting groups, with the midline shifting to the left side (Fig. 2a). As shown in Fig. 2b, brain water content was quantified by wet and dry weight method, and the result showed that the relative content started to increase in the ischemic hemisphere after pMCAO compared to the sham group at 24 h (80.85 ± 1.40% vs. 78.58 ± 0.49%, **P < 0.01), and remained stable for the next 2 days (48 h: 82.07 ± 1.79%, *P < 0.05; 72 h: 82.09 ± 1.44%, **P < 0.001). Bloodletting puncture at HTWP significantly ameliorated the water content at 24 h (78.90 ± 0.61%, ^**##**^ *P* < 0.01), 48 h (79.04 ± 0.83%, ^**#**^ *P* < 0.05) and 72 h (79.04 ± 1.03%, ^**##**^ *P* < 0.01) compared with pMCAO group.

### Bloodletting puncture at HTWP prevented the BBB disruption

Evans blue extravasation was adopted to reflect the integrity of BBB so as to evaluate the effectiveness of bloodletting puncture at HTWP in both early and late phases (5 h, 24 h and 72 h) of cerebral edema after pMCAO surgery.

**Fig. 3:**
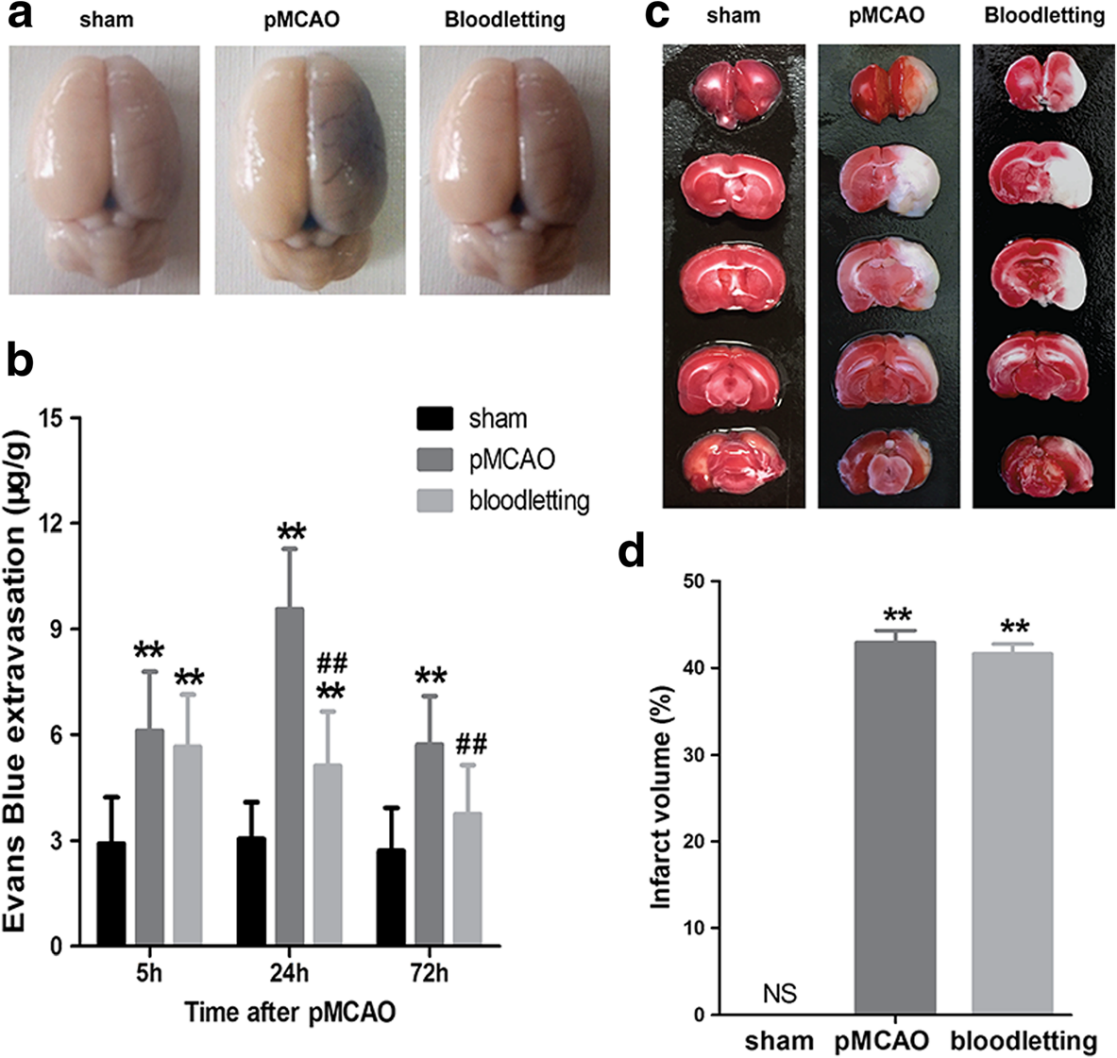
Effect of bloodletting puncture at HTWP on blood-brain barrier leakage at 5 h, 24 h, 72 h (*n* = 10 per group). **a** Representative images of brain slices stained by EB in sham-operated group, pMCAO group, bloodletting group. **b** Quantitative analysis of EB leakage. *P < 0.05, **P < 0.01 vs. sham-operated goup. ^#^ *P* < 0.05, ^##^ *P* < 0.01 vs. pMCAO group at the same time points. **c** Representative images of brain slices stained by TTC in sham-operated group, pMCAO group, bloodletting group. **d** Quantitative analysis of cerebral infarct area by TTC staining. **P < 0.01 vs. sham-operated group

As shown in Fig. 3, there was no visible staining of the brain in sham-operated controls. In pMCAO group, the extravasation of EB was observed increase in the ischemic hemisphere at 5 h (Fig. 3b, 2.91 ± 1.33 vs. 6.11 ± 1.68 μg/g, **P < 0.001), 24 h (Fig. 3a-b, 3.05 ± 1.05 vs. 9.56 ± 1.71 μg/g, **P < 0.001) and 72 h (Fig. 3a-b, 2.71 ± 1.22 vs. 5.72 ± 1.38 μg/g, **P < 0.001) compared with sham group. Bloodletting puncture at HTWP significantly inhibited the EB extravasation at 24 h (5.12 ± 1.54 vs. 9.56 ± 1.71 μg/g, ^**##**^ *P* < 0.001) and 72 h (3.76 ± 1.39 vs. 5.72 ± 1.38 μg/g, ^**##**^ *P* < 0.01) compared to pMCAO group, without any difference at 5 h. As shown in Fig. 3c-d, bloodletting puncture slightly reduced the cerebral infarct area compared with pMCAO group but without significant difference. These data demonstrated that bloodletting puncture at HTWP remarkably alleviated the impairment of the BBB following cerebral ischemia.

### Effect of bloodletting puncture at HTWP on ultrastructure alteration of BBB

**Fig. 4:**
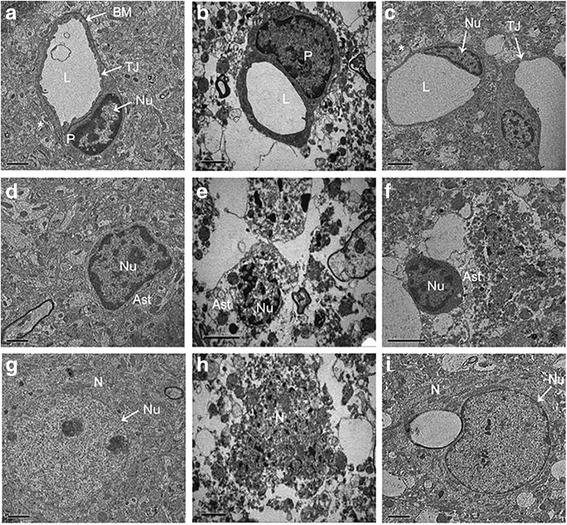
Effect of bloodletting puncture at HTWP on ultrastructure of the BBB in the ipsilateral cortex at 48 h following pMCAO (*n* = 3 per group). **a**, **d**, **g** Sham-operated group. Normal basement membrane and tight junctions were observed. **b**, **e**, **h** pMCAO group. The basement membranes were disrupted, tight junctions were unclear, and astrocytes were degenerated. **c**, **f**, **i** Bloodletting group. Continuous tight junctions and basement membrane were observed. EC, endothelial cell; L, capillary lumen. BM, basement membrane; P, pericyte; *, astrocyte end-feet; TJ, tight junctions; Ast, astrocyte; N, neuron; Nu, nucleus. Scale bar = 1 μm in (**a**, **d**, **g**, **i**). Scale bar = 2 μm in (**b**, **c**, **e**, **f**, **h**)

48 h after pMCAO surgery, the rat brains were removed and the ultrastructure of BBB was analysed with the help of transmission electron microscopy, and relevant profiles were shown in Fig. 4. The cortex capillaries in sham-operated rats showed normal ultrastructure, where the single layer of endothelial cells (ECs) of microvessels was smooth, abundant with intact tight junction (TJ) complexes, surrounded by complete basement membrane (BM), pericyte and astrocyte end-feet (Fig. 4a). The astrocytes (Fig. 4d) and neurons (Fig. 4g) demonstrated normal morphology. Compared with sham-operated group, the capillary ultrastructure in ipsilateral cortex of pMCAO rats displayed varied abnormalities (Fig. 4b, e, h), including edematous ECs and pericyte, disrupted BMs, unclear TJs (Fig. 4b), and degenerated even dissolved astrocyte (Fig. 4e) and neurons (Fig. 4h). In the bloodletting group, astrocytes (Fig. 4f) and neurons (Fig. 4i) were slightly shrunken, while continuous TJs and BMs were observed although the electron density of BMs was low (Fig. 4c).

### Bloodletting reversed the reduction of occludin and claudin-5 and inhibited the expression of ICAM-1 and VEGF

**Fig. 5:**
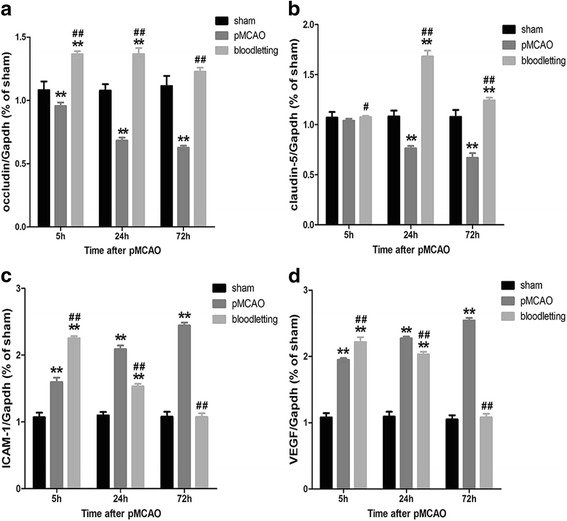
Effect of bloodletting puncture at HTWP on the mRNA expression of occludin, claudin-5, ICAM-1 and VEGF, which was detected by RT-qPCR at indicated time points (5 h, 24 h, and 72 h) (n = 10 per group). **a** The mRNA level of occludin. **b** The mRNA level of claudin-5. **c** The mRNA level of ICAM-1. **d** The mRNA level of VEGF. *P < 0.05, **P < 0.01 vs. sham-operated goup. ^#^ *P* < 0.05, ^##^ *P* < 0.01 vs. pMCAO group at the same time points

To investigate the mechanism of BBB disruption further, we examined the mRNA expression of two major proteins involved in tight junctions of the BBB, which are occludin and accludin-5 in cerebral cortex of ischemic penumbra. As showed in Fig. 5a and b, the mRNA expression levels of occludin were decreased significantly in pMCAO group at 5 h (0.96 ± 0.03 vs. 1.08 ± 0.07, **P < 0.001), 24 h (0.68 ± 0.02 vs. 1.08 ± 0.05, **P < 0.001) and 72 h (0.63 ± 0.02 vs. 1.11 ± 0.08, **P < 0.001) compared with sham group. The mRNA expression levels of claudin-5 decreased at 24 h (0.76 ± 0.02 vs. 1.08 ± 0.06, **P < 0.001) and 72 h (0.67 ± 0.05 vs. 1.08 ± 0.07, **P < 0.001). Bloodletting puncture at HTWP significantly increased the mRNA expression levels of occludin (1.37 ± 0.02, 1.37 ± 0.05, 1.23 ± 0.03, ^**##**^ *P* < 0.001 vs. pMCAO group) and claudin-5 (1.07 ± 0.01, 1.68 ± 0.06, 1.24 ± 0.03, ^**#**^ *P* < 0.05, ^**##**^ *P* < 0.001 vs. pMCAO group) at the observation time points.

As shown in Fig. 5c and d, after pMCAO, the mRNA expression levels of ICAM-1 (1.60 ± 0.07, 2.09 ± 0.06, 2.44 ± 0.04, ^**^ *P* < 0.001) and VEGF (1.95 ± 0.03, 2.27 ± 0.03, 2.54 ± 0.04, ^**^ *P* < 0.001) were observed increase significantly compared with those in sham-operated group. Bloodletting puncture at HTWP significantly up-regulated the mRNA expression of ICAM-1(2.25 ± 0.03, ^**##**^ *P* < 0.001) and VEGF (2.22 ± 0.08, ^**##**^ *P* < 0.001) at 5 h, and down-regulated the mRNA level of ICAM-1 (1.53 ± 0.04, 1.08 ± 0.05, ^**##**^ *P* < 0.001) and VEGF(2.03 ± 0.04, 1.08 ± 0.05, ^**##**^ *P* < 0.001) at 24 h, 72 h compared with those in pMCAO group.

## Discussion

pMCAO is a classical model to study the mechanisms of cerebral ischemic damage [13]. Brain edema is an important factor to the high morbidity and mortality of large-scaled ischemic strokes and occurs due to the accumulation of excess water in the brain parenchyma [15]. In current study, we found that bloodletting puncture at HTWP could alleviate brain swelling ever since 24 h after ischemia. The underlying mechanisms may be related to tight junction and endothelial protection via up-regulating the expression of occludin and claudin-5 and down-regulating ICAM-1 and VEGF expression.

Bloodletting puncture at HTWP has been applied as one of first aid measures in various types of emergency for more than 3000 years in China. To access whether this traditional therapy plays neuro-protective role against central nervous injury, we previously demonstrated the bloodletting puncture therapy could substantially alleviate conscious disturbance, cerebral edema and neurological deficits in pMCAO and traumatic brain injury patients or animal models [7–10, 12]. Taking current studies into consideration, bloodletting puncture at HTWP could be a safe, effective, and easily-manipulated neuro-protective therapy in the treatment of central nervous injuries, with potential to be developed into a pre-hospital first aid treatment.

In order to clarify the mechanism of neuron and BBB protective effects, our group and colleagues have carried out serious of researches. Firstly, we demonstrated that bloodletting therapy could inhibit the accumulation of H^+^ and extracellular Ca^2+^ movement as well as alleviate intracellular Ca^2+^ overload state in ischemic region of ischemic animal models [16, 17]. Secondly, this therapy could restore local content of K^+^ and Na^+^ which might prevent the occurrence of brain edema [18]. Thirdly, bloodletting increased the cerebral blood flow in ischemic patients and rabbits which might improve local micro-circulation and prevent neuron apoptosis [19, 20]. On the peripheral signal transduction pathway, the contribution of three factors including bleeding, peripheral nerve conduction and specificity of *Jing*-well points were evaluated by using ischemic rabbit models [21]. The results indicated that bleeding and *Jing*-well acupoints were necessary for this therapy as the enhancement of cerebral blood flow was greater in bleeding group and *Jing*-well groups when compared with non-bleeding and Quchi acupoint (LI 11) groups, respectively [21, 22]. Meanwhile, when the radial, ulnar and median nerves were sectioned in the ischemic rabbit models, bloodletting therapy could not enhance the cerebral blood flow [21], indicating these nerves played an important role in signal transduction from *Jing*-well acupoints. Taking current studies into consideration, we further clarified the molecular mechanism by which bloodletting puncture therapy attenuated ischemic stroke induced cerebral edema via regulating BBB dysfunction.

Usually brain edema includes two types: cytotoxic and vasogenic. Cytotoxic edema is the intracellular accumulation of water due to energy failure and inability of cells to regulate their volumes (starting from within 1 h after ischemia) [15]. Vasogenic edema mainly involves in the disruption of the BBB, usually occurring no earlier than 3 h since beginning, and continuously developing in the following 24–72 h in ischemic rats [11]. Our previous studies have shown bloodletting puncture therapy did not affect the brain edema within 5 h, and the current study has confirmed bloodletting could ameliorate vasogenic brain swelling within 24–72 h, but not targeting the cytotoxic edema.

Using EB and TTC staining, we characterized the topographic distribution of ischemic BBB damage and tissue injury at 48 h following pMCAO, and found bloodletting puncture slightly reduced the cerebral infarct area compared with pMCAO group but without significant difference. The results partly support the improvement of tight junction and cerebro-microvasculature is main target of bloodletting but not direct neuro-protection. Brain edema could not be rescued at 5 h when tissue injury became serious according to previous and present studies, thus the infarct area was improved on some extent at 48 h when the BBB damage was ameliorated. This is in line with the general belief that the neuronal tissue is more susceptible to ischemic insult than the cerebro-microvasculature [23].

The BBB is a unique and integral feature of the central nervous system, regulated by endothelial cells, pericytes and the end-feet of astrocytes [24, 25]. The main cause of vasogenic brain edema is the dysfunction and opening of BBB. However, BBB opening is heterogeneous depending upon the nature of stroke. Extensive research in transient occlusion and reperfusion models suggests that BBB compromise after stroke occurs in two phases: an immediate early phase of enhanced permeability seen 4–6 h after ischemia followed by a delayed opening of the BBB seen 2–3 days after stroke [15]. While the time course of BBB opening differs in permanent occlusion from several hours to 48 h, it still showed an irreversible opening of the BBB with prolonged duration of ischemia [26, 27]. According to the BBB opening type in pMCAO model which we investigated, pMCAO brain became swelling at 24 h due to the BBB irreversible opening, while BBB increased permeability started from 5 h. What was more important was that bloodletting therapy could ameliorate the brain swelling since 24 h and prevent the BBB broken from 5 h.

The intact tight junction between brain capillary cells is critical to normal brain barrier function in reducing the permeability of cerebral vessels and restricting the free molecular exchange between blood and brain tissues, for which reason, structural damage of tight junctions could cause the leakage of BBB and brain edema [28]. We found bloodletting therapy could benefit tight junction structure including endothelial cells, basement membrane and astrocyte end-feet under electron microscope. In order to explore the molecular regulatory targets on tight junctions by bloodletting, several protein components associated with tight junction were investigated in present study. Occludin was the first tight junctional transmembrane molecule discovered [29]. It has been demonstrated that all the external loops, transmembrane and C-terminal cytoplasmic domains of occludin are important to the regulation of paracellular permeability [30, 31]. The N-terminal cytoplasmic domain of occludin regulates transepithelial migration of neutrophils, whose process is independent of the transepithelial resistance and the paracellular permeability [31]. Meanwhile, occludin was found responsible for the sealing of tight junctions [32]. Claudins are integral membrane proteins which share the four transmembrane domains of occludin, without containing any sequence homology to occludin. The only claudins detected in endothelial cells thus far are claudin-1 and claudin-5 [33–36]. We used *Claudin-5:eGFP* transgenic mice to visualize endothelial TJs, ultrastructural changes in TJ assemblies such as discontinuous TJs and extensions from linear TJ strands [37]. In current study, we found the regulatory target of BBB for bloodletting therapy might be these molecules on tight junctions.

The result of the study revealed that VEGF expression in pMCAO brain of rats was increased from 5 h to 72 h in accordance to previous studies [38–40]. It was reported that, in chronic phase of ischemic stroke, VEGF can promote angiogenesis [41]. On the other condition, however, VEGF can also serve as potent vascular permeability factor which increases the permeability of microvascular towards blood plasma proteins in early stage [41]. Thus the up-regulation of VEGF is closely associated with BBB disruption. Our results also found VEGF was inhibited by bloodletting which might be involved in the improvement of brain edema, but the action of bloodletting in late stage of pMCAO needs further confirmation.

ICAM-1 is the best characterized cell surface adhesion molecule constitutively expressed on brain microvascular endothelial cells. Adhesion of leukocytes to endothelium mediated by ICAM-1 has been considered to be closely related to the pathological processes of immune-mediated central nervous diseases [42]. The protective effect of bloodletting therapy on endothelial cells is also supported by its inhibition on the ICAM-1 expression levels at acute, regulatory changes in barrier function, as demonstrated in this study. In present study, the level of ICAM-1 and VEGF showed greater upregulation at 5 h after modeling without any effective influence on brain edema. We speculated the upregulation was attributed to a compensation or a stress response immediately after bloodletting puncture. And we speculated that bloodletting puncture may alleviate the brain edema partly via immune modulation and related neuro-immune mechanism will be conducted in future studies.

## Conclusions

In conclusion, our results illustrated that bloodletting puncture at HTWP was effective for attenuating the extent of brain edema formation in response to ischemia injury in rats, partly by protective effect on the tight junction of BBB, highlighting a safe, effective, and easily-manipulated neuro-protective therapy in ischemic stroke.

## Abbreviations

BBB: blood-brain barrier
BM: Basement membrane
EB: Evans blue
ECs: Endothelial cells
HTWP: Hand twelve *Jing*-well points
pMCAO: Permanent middle cerebral artery occlusion
RT-qPCR: Real-time quantitative PCR
TBI: Traumatic brain injury
TEM: Transmission electron Microscopy
TJ: Tight junction
